# Using Chitosan-Coated Polymeric Nanoparticles-Thermosensitive Hydrogels in association with Limonene as Skin Drug Delivery Strategy

**DOI:** 10.1155/2022/9165443

**Published:** 2022-04-07

**Authors:** Estefânia V. R. Campos, Patrícia L. F. Proença, Tais G. da Costa, Renata de Lima, Leonardo F. Fraceto, Daniele R. de Araujo

**Affiliations:** ^1^Human and Natural and Sciences Center, Federal University of ABC, Santo André, SP, Brazil; ^2^Drugs and Bioactives Delivery Systems Research Group-SISLIBIO, Federal University of ABC, Brazil; ^3^São Paulo State University (UNESP), Laboratory of Environmental Nanotechnology, Institute of Science and Technology of Sorocaba, Sorocaba, SP, Brazil; ^4^LABiToN (Laboratory of Bioactivity Assessment and Toxicology of Nanomaterials), University of Sorocaba, Sorocaba, SP, Brazil

## Abstract

Topical delivery of local anesthetics (LAs) is commonly used to decrease painful sensations, block pain throughout procedures, and alleviate pain after surgery. Dermal and/or transdermal delivery of LAs has other advantages, such as sustained drug delivery and decreased systemic adverse effects. This study reports the development of poly(D,L-lactide-co-glycolide) (PLGA) nanoparticles coated with chitosan for the sustained release and topicality of benzocaine (BZC) and topical delivery. BZC PLGA nanoparticles or nonencapsulated drugs were further incorporated into Poloxamer hydrogels (Pluronic™ F-127). The nanoparticles showed a mean diameter of 380 ± 4 nm, positive zeta potential after coating with chitosan (23.3 ± 1.7 mV), and high encapsulation efficiency (96.7 ± 0.02%). Cellular viability greater than 70% for both fibroblasts and keratinocytes was observed after treatment with nanoparticles, which is in accordance with the preconized guidelines for biomedical devices and delivery systems. Both the nanoparticles and hydrogels were able to modulate BZC delivery and increase drug permeation when compared to the nonencapsulated drug. Furthermore, the incorporation of limonene into hydrogels containing BZC-loaded nanoparticles increased the BZC permeation rates. Non-Newtonian and pseudoplastic behaviors were observed for all hydrogel nanoformulations with or without nanoparticles. These results demonstrate that the hydrogel-nanoparticle hybrids could be a promising delivery system for prolonged local anesthetic therapy.

## 1. Introduction

Topical delivery of local anesthetics (LAs) has gained much attention as it allows for sustained drug delivery, fewer systemic adverse effects, easy application, and increased patient compliance [1]. LAs are drugs that reversibly block neural transmission by inhibiting excitation conduction in peripheral nerves [2, 3]. Topical anesthesia is widely used to reduce or alleviate pain in dentistry procedures, in procedures requiring local anesthetic injections, and after operation [4]. These medications can also be used to decrease pain or discomfort due to skin irritation, sunburns, and several other causes of minimal discomfort on the body surface. Topical anesthesia is widely used on mucous membranes, such as the nose, throat, rectum, and vagina, to minimize the pain of some medical procedures, such as the insertion of tubes or a speculum [5]. In addition, topical anesthesia is widely used in invasive airway procedures such as endoscopy, intubation, and bronchoscopy [6]. Long-lasting local anesthesia is preferred to control postoperative pain, usually achieved by the association of LAs with various drug delivery systems [3, 7].

Benzocaine (BZC) is a poorly soluble ester-type LA that is useful for topical (dermal and mucosal) applications. Its latency time is rapid (<1 min) with an effective duration of approximately 5–10 min, which is short compared to the potential pain duration [8]. Topical applications are usually nontoxic; however, due to their short duration, increased dosages are required, often resulting in severe side effects. For example, when a high concentration of BZC in plasma is metabolized, it generates a toxic metabolite called PABA, which is responsible for causing methemoglobinemia [9]. Thus, the development of drug delivery systems capable of modulating the BZC release rate, decreasing its systemic absorption and consequently toxicity, and improving its skin retention time could be very promising [10]. Different types of nano-based carrier systems, such as liposomes, polymeric nanoparticles, solid lipid nanoparticles, nanostructured lipid carriers, and cyclodextrin, have been studied for dermal LA delivery [10].

A previous report studied the encapsulation of BZC into polymeric nanoparticles composed of different polymers, such as poly(*ε*-caprolactone) (PCL), poly(L-lactide) (PLA), and poly(D,L-lactide-co-glycolide) (PLGA) (2012) [11]. According to this study, PLA nanoparticles were able to prolong BZC anesthetic action and exhibited the greatest potential for application [11]. Although the suspension of nanoparticles is easy to apply, the topical suspension of nanoparticles tends to agglomerate, separate, and coalesce, which may result in nonuniform distribution of the nanoparticles on the skin surface [12]. In addition, these nanoformulations do not have adequate viscosity, which results in the suspension flowing from the application site [13]. To overcome these limitations, the development of hybrid carrier systems that associate nanoparticles with hydrogels is an attractive alternative [13–15].

PLGA is approved by the Food and Drug Administration (FDA) and the European Medicine Agency (EMA) as a biodegradable and biocompatible polymer for drug delivery in humans. When PLGA is hydrolyzed and biodegraded, it generates lactic and glycolic acids, which are metabolized by the body via the Krebs cycle, which may result in minimal systemic toxicity [16]. High-viscosity, water-soluble carriers are widely used to obtain sustained-release nanoformulations. Among them, Pluronic F-127 (Poloxamer 407) has been extensively used for topical delivery because of its unique properties including low toxicity, slow sustained drug release, thermoreversible gelation, and nontoxicity [17]. The most interesting property of the Poloxamer P407-based topical nanoformulation is its reverse thermal gelation, where at lower temperatures, it shows liquid-like behavior and becomes semisolid as the temperature increases. Depending on the polymer concentration, the nanoformulation exhibits a transition temperature (*T*_sol–gel_) below or close to the human physiological body temperature [17].

Chitosan (CS) is the second most abundant natural polymer obtained from the alkaline deacetylation of chitin [18]. This cationic amino-polysaccharide has several advantages, specifically biocompatibility, biodegradability, and pH sensitivity; it is soluble in water up to pH 6 and insoluble at basic pH [18, 19]. CS is widely used in delivery systems because of its mucoadhesive and antimicrobial properties as well as its permeation-enhancing effects [20]. An interesting property of CS is that it increases the permeability of actives by inducing the transient opening of junctions located in cell membranes [21, 22].

Permeation promoters are substances incorporated into the topical nanoformulations, resulting in a reversible increase in permeability. They can interact with constituents of the stratum corneum, thus reducing the skin's resistance to drug permeation, or they can increase drug flux due to an increase in the thermodynamic activity of the nanoformulations [23]. Terpenes are the main constituents of essential oils and have low skin irritation when used at low concentrations (<5%). They are recognized as skin permeation enhancers and thus are increasingly becoming of interest to the pharmaceutical industry [24]. Limonene (LIM) is a monocyclic unsaturated terpene that is the major compound in many citrus (orange and lemon) oils. Previous studies have shown that limonene has increased permeability to different molecules, and when compared to other terpenes and some synthetic permeators, such as Span 20 and oleic acid, it has superior activity [25–27].

Given the above findings, this study sought to facilitate the permeation of LAs through topical application by developing PLGA nanoparticles coated with chitosan. We produced this nanoformulation using the emulsification/evaporation method and then dispersed it into P407-based hydrogels alone or associated with limonene, a penetration enhancer. All nanoparticles were characterized by mean diameter, polydispersity index, zeta potential, morphology, and drug encapsulation efficiency. In addition, the rheological properties of the hydrogels with or without nanoparticles in their matrices were evaluated. BZC release and permeation were evaluated using Franz cells employing cellulose and StratM® membranes, respectively, and the MTT assay was used to evaluate the cellular viability of fibroblasts and keratinocytes exposed to different concentrations of nanoparticles.

## 2. Materials and Methods

### 2.1. Chemicals and Reagents

Pluronic F127® (P407), poly(D,L-lactide-co-glycolide) (PLGA), benzocaine (BZC), and poly(vinyl alcohol) (PVA) were purchased from Sigma-Aldrich and used without further purification. Myritol 318 was purchased from ChemSpecs. Acetonitrile and methanol (HPLC grade) were obtained from J.T. Baker. Acetone, dichloromethane, and ethanol were purchased from local suppliers.

### 2.2. Preparation of Nanoparticles

BZC-loaded nanoparticles were prepared using the emulsification/evaporation technique adapted from Zhou et al. Briefly, the organic phase was composed of 100 mg PLGA, 20 mL methylene chloride, 10 mL acetone, 100 mg Myritol 318, and 40 mg BZC. The organic phase was emulsified with 50 mL of an aqueous solution containing 3 mg/mL PVA and 10 mL ethanol using an Ultraturrax (14000 rpm) for 8 min. Subsequently, the organic solvent was removed under reduced pressure using a rotary evaporator to obtain a final volume of 5 mL. The resultant colloidal dispersion was mixed with a 5 mL chitosan solution (0.5% *w*/*v*) and was stirred for 1 h. To remove unreacted chitosan, the nanoparticle suspension was centrifuged at 23.813 g, and the pellet was resuspended in distilled water. This process was repeated thrice. The final concentration of BZC in the nanoformulation was 0.4% [28].

### 2.3. Hydrogel Preparation Containing Nanoparticles

Poloxamer 407 hydrogels were prepared using the cold method [29]. For the preparation of the hydrogel, a 100 mL stock solution (40% *w*/*v*) was prepared by dissolving the polymer in ultrapure water with a magnetic stir bar. This solution was placed in an ice bath for approximately 8 h until complete polymer dissolution and transparency. After polymer dissolution, the hydrogels were refrigerated at 4°C for 12 h. For the preparation of the hydrogel containing nanoparticles, a 7.5 mL hydrogel stock solution (40% *w*/*v*) was magnetically stirred with either a 2.5 mL PLGA nanoparticle solution containing BZC or nonencapsulated BZC in an ice bath for 2 h. For the hydrogels containing penetration enhancers, different concentrations (0.5, 0.75, and 1% *v*/*v*) of limonene were magnetically stirred with the hydrogel solution prior to the addition of nanoparticle solution. The final concentrations of Poloxamer 407 and BZC in the hydrogels were 30% *w*/*v* and 0.1% *w*/*v*, respectively. Hydrogels containing control nanoparticles (no BZC) and control hydrogels (no nanoparticles) were prepared using the same volumes as described above.

### 2.4. Nanoparticle Physicochemical Characterization

#### 2.4.1. Size Distribution, Zeta Potential, and Morphological Analysis

Dynamic light scattering and microelectrophoresis were used to evaluate the nanoparticle size, polydispersity index, and zeta potential. Samples were diluted 100-fold, equilibrated at 25°C for 120 s, and analyzed at a fixed angle of 173° using ZetaSizer HSA 300 (Malvern®). In addition, the nanoparticle size and concentration were evaluated using nanoparticle tracking analysis (NTA). The samples were diluted 10.000-fold and analyzed using a NanoSight LM10 cell (green laser, 532 nm) and an sCMOS camera controlled by NanoSight v. 3.1. Data are expressed as the average of three measurements. The morphology of the nanoparticles was evaluated using scanning electron microscopy (SEM; FEI-Inspect-F50) available at LNNano, Campinas. A drop of the nanoparticles was placed onto a previously treated silicon substrate (glow discharge). The samples were then coated with 10 nm carbon in an argon atmosphere. The SEM microscope was operated at 2 kV with a spot size of 3 and a work distance of approximately 10 mm.

#### 2.4.2. Benzocaine Encapsulation Efficiency and Drug Loading Capacity

The encapsulation efficiency of BZC in PLGA nanoparticles was evaluated using an ultrafiltration/centrifugation method employing ultracentrifugation devices with 10 kDa MWCO (Amicon, Millipore). After filtration, the nonencapsulated BZC in the filtrate was quantified by high-performance liquid chromatography (HPLC) using an Ultimate 3000 instrument (Thermo Fisher Scientific, Waltham, USA) with Chromeleon 7.2 software for acquisition and analysis of the chromatogram. The mobile phase was comprised of methanol : water (60 : 40 *v*/*v*), and we employed a Phenomenex C18 column (250 × 4.60 mm, 5 *μ*m) [30]. Linear regression was applied to the points of the calibration curves, resulting in the following equation: *y* = 10.508*x* + 0.5712. The drug loading capacity (LC%) was calculated as the amount of the total drug encapsulated divided by the total nanoparticle weight.

### 2.5. Rheological Studies

All rheological studies of the hydrogels were performed using an oscillatory rheometer (Kinexus Lab, Malvern Instruments Ltd., UK) equipped with cone-plate geometry. All hydrogels were subjected to oscillatory measurements, and the flow properties were determined. Frequency sweep analyses were carried out in the frequency range of 0.1 to 10 Hz, and the temperature was kept constant at 32.5°C. For temperature sweep analysis, the frequency was maintained at 1 Hz, and the temperature ranged from 10 to 50°C. The oscillatory measurements were utilized to calculate the elastic module (*G*′), viscous module (*G*^″^), viscosity (*ƞ*), and sol–gel temperature transition for each nanoformulation. Data were analyzed using GraphPad Prism 8. The flow properties were evaluated with shear rates ranging from 0.1 to 500 s^−1^ with a ramp time of 120 s. Data were analyzed using GraphPad Prism 8.

### 2.6. *In Vitro* Release Assays and Mathematical Modeling

*In vitro* release profiles of encapsulated benzocaine (NP_BZC), emulsified benzocaine nonencapsulated (EM_BZC), hydrogels containing benzocaine encapsulated (NP_BZC_P407) or nonencapsulated (EM_BZC_P407), and hydrogels containing benzocaine encapsulated with limonene (0.5–1% *v*/*v*) (NP_BZC_P407_LIM 0.5%, NP_BZC_P407_0.75%, and NP_BZC_P407_1%) were evaluated using a two-compartment system under sink conditions. This model is composed of a 1 mL donor compartment and a 100 mL acceptor compartment, separated by a cellulose membrane with a molecular exclusion pore size of 1 kDa. At predetermined times, 1 mL aliquots were withdrawn from the acceptor compartment, and the concentration of BZC was determined by HPLC. To maintain the same volume of the medium throughout the experiment, the volume withdrawn was replaced with fresh phosphate buffer (0.1 M, pH 7.4) at 32.5°C. Zero-order, first-order, Higuchi, and Korsmeyer-Peppas mathematical models were applied to elucidate the BZC release mechanism in all nanoformulations.

### 2.7. *In Vitro* Permeation Studies Using Strat-M® Artificial Membrane

*In vitro* permeation kinetics tests of encapsulated benzocaine (NP_BZC), emulsified benzocaine nonencapsulated (EM_BZC), hydrogels containing benzocaine encapsulated (NP_BZC_P407) or not encapsulated (EM_BZC_P407), and hydrogels containing benzocaine encapsulated with limonene (0.5-1% *m*/*v*) (NP_BZC_P407_LIM 0.5%, NP_BZC_P407_0.75%, and NP_BZC_P407_1%) were performed using a vertical cell Franz apparatus (Microette Plus; Hanson Research, Chatsworth, CA, EUA), which is a two-compartment system, composed of one 1 mL donor compartment (permeation area 1.72 cm^2^) and a 7 mL acceptor compartment separated by an artificial membrane Strat-M® (25 mm disc—Millipore Co, USA), which simulates skin models [13, 31]. The acceptor compartment was filled with a 0.1 M phosphate buffer solution containing 154 mM NaCl, at pH 7.4, under constant stirring, and the temperature was maintained at 32.5 ± 0.5°C. At predetermined time intervals, 1 mL aliquots were withdrawn from the acceptor compartment, and the concentration of BZC was determined by HPLC. To maintain the same volume in the acceptor compartment throughout the experiment, the volume withdrawn was replaced with a fresh phosphate buffer solution. BZC permeation was expressed as *μ*g/cm^2^ and plotted as a function of time (h) [32]. The permeation parameters, such as drug flux through the membrane (*J*, % cm^−2^·h^−1^), permeability coefficient (*P*, cm·h^−1^), and lag time (h), were calculated through linear regression of the permeation curve (linear region) using

(1) $$\begin{array}{c} J=P\cdot Cd, \end{array}$$

where *J* is the drug flux through the skin, *P* is the permeability coefficient, and *Cd* is the drug concentration in the donor compartment [33].

### 2.8. *In Vitro* Cytotoxicity Assays

The cytotoxic effects of control nanoparticles (NP_CTL), encapsulated benzocaine (NP_BZC), and emulsified benzocaine nonencapsulated (EM_BZC) were evaluated by measuring the metabolic activity of the cells using a colorimetric method. This method is based on the reduction of MTT, a yellow water-soluble tetrazolium dye, to a purple compound by viable cells. The cytotoxicity of the different nanoformulations was evaluated in two cell lines: keratinocytes (HaCaT) and fibroblasts (3T3). The cell lines were maintained in continuous culture using a DMEM culture medium, supplemented with 10% fetal bovine serum, 100 UI/mL penicillin, and 100 *μ*L streptomycin sulfate, at pH 7.4, 37°C, and 5% CO_2_. Cells were seeded in 96-well culture plates at a concentration of 1 × 10^4^ viable cells per well and incubated for 24 h. The cells were then exposed to either encapsulated or nonencapsulated BZC for 24 h at concentrations ranging from 0 to 650 *μ*M. The control nanoparticles were tested at the same dilution used for the BZC-loaded nanoparticles.

## 3. Results and Discussion

### 3.1. Nanoparticle Physicochemical Characterization and Colloidal Stability

**Figure 1:**
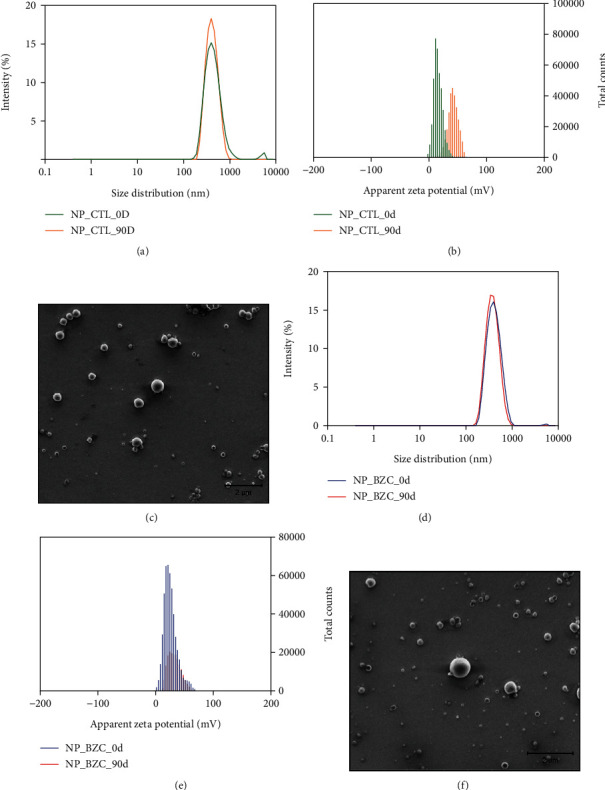
Physicochemical characterization of nanoparticles: (a, d) mean diameter of control nanoparticles (NP_CTL) at time 0 (green lines) and after 90 days (orange lines) and BZC-loaded nanoparticles (NP_BZC) at time 0 (blue lines) and after 90 days (red lines) determined using DLS. (b, e) The zeta potential of the control nanoparticles (NP_CTL) and BZC-loaded nanoparticles (NP_BZC). (c, f) SEM images captured of the control nanoparticles (NP_CTL, 29.365x magnification) and BZC-loaded nanoparticles (NP_BZC, 23.646x magnification).

Nano-based drug delivery systems have been extensively studied to improve the therapeutic effects of drugs because of modifications in their biodistribution profile and pharmacokinetics [10]. In addition, the association of these systems with hydrogels, to form a hybrid biomaterial, increases the therapeutic index of nano-based drug delivery systems [13, 14]. We prepared chitosan-coated PLGA nanoparticles loaded with BZC using the emulsification/evaporation method; these nanoparticles were then added to Poloxamer P407 hydrogels. The physicochemical properties and colloidal stability of the control nanoparticles (NP_CTL) and BZC-loaded nanoparticles (NP_BZC) were evaluated as a function of storage time (up to 90 days). The physicochemical properties, such as mean diameter and zeta potential after preparation and 90 days of storage, as well as the morphological characterization of both NP_CTL and NP_BZC are summarized in Figure 1.

The initial mean diameters of the control nanoparticles (Figure 1(a), green line) and BZC-loaded nanoparticles (Figure 1(d), blue line), measured by DLS, were 331 ± 6 nm and 380 ± 4 nm, respectively. For both nanoparticles, changes were observed by examining the mean size as a function of storage time. However, it is worth mentioning that no significant differences in size were found between the initial 0 timepoint and after 90 days of storage for both nanoformulations studied, as shown in Figures 1(a) and 1(d) for the control and BZC-loaded nanoparticles, respectively. Both NP_CTL and NP_BZC showed a unimodal size distribution with polydispersity indices of 0.098 ± 0.006 and 0.136 ± 0.05, respectively.

The mean diameter was also measured using nanoparticle tracking analysis (NTA) for both NP_CTL and NPC_BZC, where the mean diameter 256 ± 5 nm and 239 ± 9 nm, respectively, which is smaller compared to the mean diameter obtained by DLS (Figure S1). Although both techniques measure the size of the nanoparticles based on their diffusion coefficient, DLS reads the modification of scattered light to determine the nanoparticle diffusion coefficient, while NTA estimates the diffusion coefficient of individual particles in different videos. There were no significant differences in the mean diameter of the nanoparticles as a function of the 90-day storage time. Additionally, NTA was used to measure the concentrations of the nanoparticles for NP_CTL and NP_BZC which were 1.57 ± 0.05 × 10^13^ particles/mL and 1.66 ± 0.09 × 10^13^ particles/mL, respectively (Figure S1). After analysis, the formation of aggregates as a function of storage time was not observed, as evidenced by the decrease in the concentration of particles.

The zeta potentials of the noncoated PLGA nanoparticles were measured and determined to be around –22.5 ± 0.4 mV, likely negative due to the presence of PLGA carboxyl groups situated on the nanoparticle surface. The negative surface charge of PLGA with or without BZC present was used to electrostatically deposit CS onto its surface. Therefore, the zeta potential of CS-coated nanoparticles was positive for the control and BZC-loaded nanoparticles (22.9 ± 0.7 and 23.3 ± 1.7 mV, respectively), as shown in Figures 1(b) and 1(e). After 90 days of storage, there was a significant decrease in the zeta potential of the control nanoparticles (NP_CTL). However, these nanoparticles were produced using PVA as a stabilizing agent, which promotes stabilization through steric impediment effects; thus, changes in the electrostatic charge are not the main stabilization mechanism for these nanoformulations. The positive charge is attributed to the amino groups present in the chitosan backbone, which suggests that the PLGA nanoparticles were successfully coated with chitosan. Different studies have used electrostatic deposition of chitosan into polymeric nanoparticles to improve the sustained-release properties and cell internalization [34, 35]. In addition, morphological investigations were performed using SEM images of the dried nanoparticles. The nanoparticles showed a spherical shape and smooth surface without aggregation. The mean diameter obtained by SEM was 143 ± 24 nm for NP_CTL (Figure 1(c)) and 125 ± 10 nm for NP_BZC (Figure 1(f)), which were smaller than those obtained by DLS and NTA because of the absence of water surrounding the nanoparticles.

Finally, the encapsulation efficiency was evaluated, which is important for reducing the dose and manufacturing costs and is directly related to desirable biological activity. High encapsulation efficiency (96.7 ± 0.02%) was achieved due to the great affinity between BZC and the Myritol 318 oily core of the PLGA nanoparticles. A significant decrease in encapsulation efficiency (84.2 ± 0.5%) was observed after 90 days of storage. The drug loading capacity obtained for PLGA containing CS nanoparticles was 10.3%. The physicochemical characteristics of PLGA nanoparticles, with or without BZC, in the present study were very close to those reported in the literature [11, 36, 37].

### 3.2. Rheological Properties: Influence of Nanoparticles and Penetration Enhancer Incorporation on the Hydrogel's Structural Organization

Evaluation of the rheological properties is an important physicochemical characterization of semisolid preparations and establishes relationships with their performance as pharmaceutical nano-based formulations. It has been well characterized that the flow characteristics of the nanoformulations affect the residence time at the administration site as well as the release rate of the encapsulated compound. The physicochemical characteristics of drugs and/or nanoparticles can affect the mechanical properties of hydrogels; thus, the effects of BZC, limonene, and nanoparticles on the rheological properties of the Poloxamer P407 hydrogel were investigated.

The rheological characterization of Poloxamer P407-based hydrogel nanoformulations was performed at 32.5°C to mimic the physiological skin surface temperature. The Poloxamer P407-based hydrogels' apparent viscosity (*η*∗) values measured at 1 Hz are summarized in Table 1. The results showed increased viscosity when BZC was added to the Poloxamer P407-based hydrogels compared with the control hydrogel (without BZC). The addition of BZC and limonene in NP_BZC and NP BZC_LIM 0.5% resulted in a slightly decreased viscosity in comparison to the viscosity of the control hydrogel, NP_CTL. However, the incorporation of 1% limonene into the hydrogels evoked an increase in the *G*′/*G*^″^ relationship, suggesting that the limonene addition better promotes the polymeric matrix structural organization than the simple hydrogel matrix. Considering that Poloxamer-based hydrogels are formed through micellar self-assembly that depends on the temperature and polymer concentration, the micelle aggregation process is followed by the dehydration of the polyoxypropylene glycol hydrophobic units [38]. Because of its hydrophobic features (log *P* ~4.6) [39], limonene is most likely incorporated into the micellar core, favoring micellar aggregation and hydrogel structural organization, as detected by the high *G*′/*G*^″^ relationship values.

**Figure 2:**
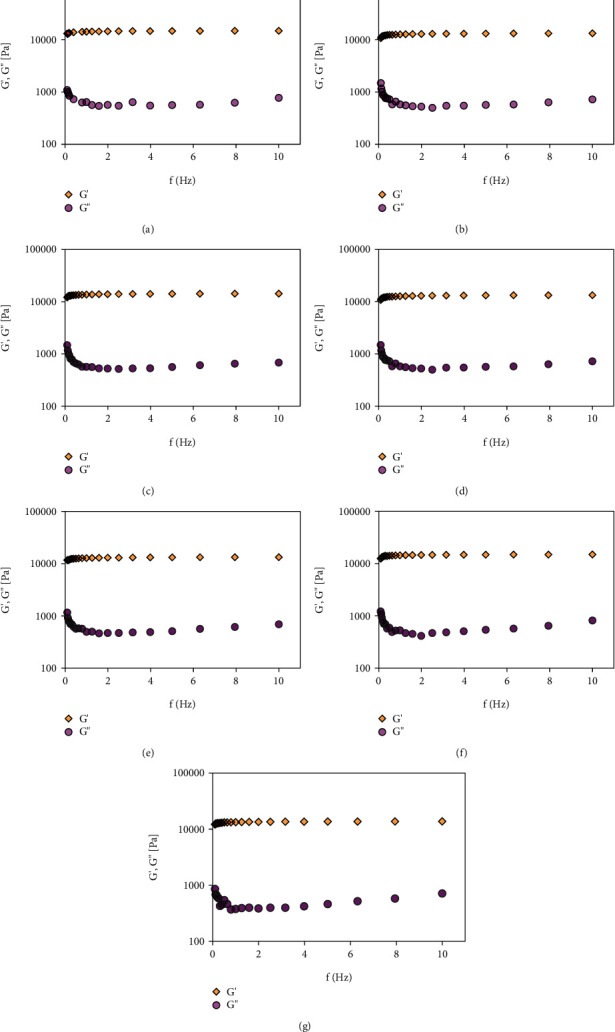
Rheological analysis of Poloxamer hydrogels (30% *w*/*v*) in function of frequency (0.1–10 Hz): (a) control hydrogel (P407–30%), (b) hydrogel containing BZC (BZC_P407), (c) hydrogel containing PLGA nanoparticles (NP CTL_P407), (d) hydrogel containing PLGA nanoparticles loading BZC (NP BZC_P407), (e) hydrogel containing PLGA nanoparticles loading BZC plus 0.5% limonene (NP BZC+LIM 0.5%_P407), (f) hydrogel containing PLGA nanoparticles loading BZC plus 0.75% limonene (NP BZC+LIM 0.75%_P407), and (g) hydrogel containing PLGA nanoparticles loading BZC plus 1% limonene (NP BZC+LIM 1%_P407).

All hydrogel nanoformulations demonstrated pseudoplastic behavior in that their viscosity decreased with high shear rate stress, resulting in low-viscosity nanoformulations, which facilitated their flow and consequently the skin spreadability as topical nanoformulations. Additionally, in the frequency sweep analysis (0.1–10 Hz) performed at 32.5°C (Figure 2), the storage moduli (*G*′) of all nanoformulations were higher than the viscous moduli (*G*′), even at lower frequencies. Furthermore, only slight variations in the *G*′ values as a function of frequency (0.1–10 Hz) were observed, indicating that all nanoformulations showed gel-like structure at 32.5°C and were stable through higher shear stress. For topical nanoformulations, pseudoplastic nanoformulations are desired as this characteristic facilitates uniform distribution onto the skin. Similar rheological profiles of Poloxamer hydrogels loaded with nanoparticles have been reported [13, 40].

**Figure 3:**
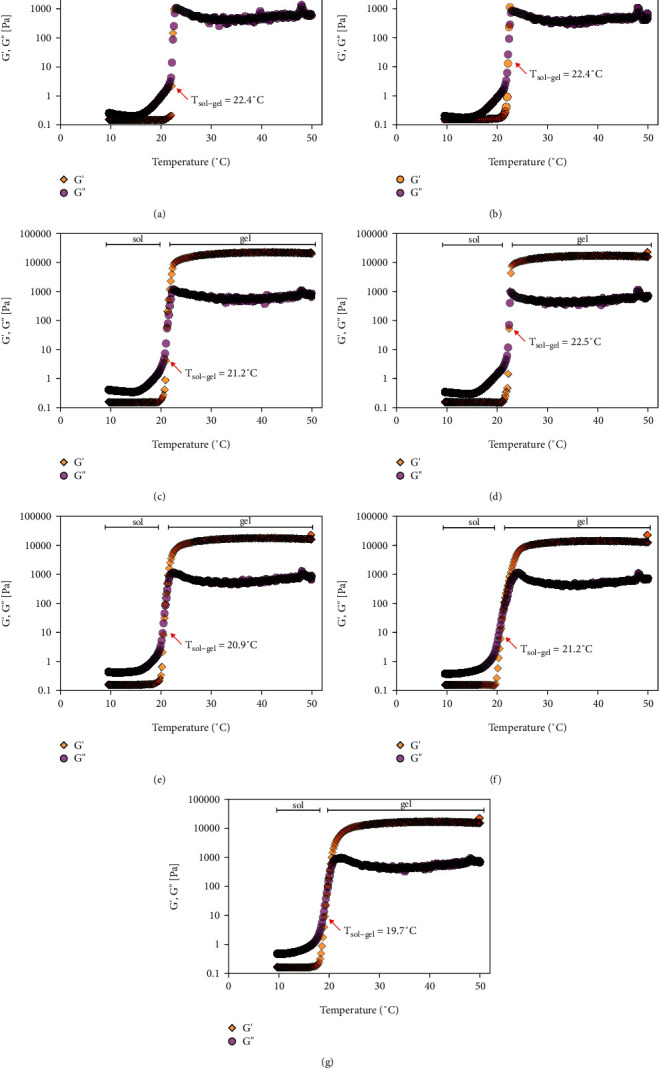
Rheological analysis of Poloxamer hydrogels (30% *w*/*v*) as a function of temperature (10–50°C): (a) control hydrogel (P407–30%); (b) hydrogel containing BZC (BZC_P407); (c) hydrogel containing PLGA nanoparticles (NP_CTL_P407); (d) hydrogel containing PLGA nanoparticles loading BZC (NP_BZC_P407); (e) hydrogel containing PLGA nanoparticles loading BZC plus 0.5% limonene (NP_BZC_P407_LIM 0.5%); (f) hydrogel containing PLGA nanoparticles loading BZC plus 0.75% limonene (NP_BZC_P407_LIM 0.75%); (g) hydrogel containing PLGA nanoparticles loading BZC plus 1% limonene (NP_BZC_P407_LIM 1%).

Poloxamer-based hydrogels are characterized by their viscoelastic properties, including a storage modulus, *G*′, which characterizes the quantity of energy stored and recovered per deformation cycle of a solid-like material. The loss modulus, *G*, represents the liquid part [41]. The influence of BZC (BZC_P407), the nanoparticle control (NP CTL_P407), BZC-loaded nanoparticles (NP BZC_P407), limonene (LIM 0.5%_P407), and BZC-loaded nanoparticles plus 0.5, 0.75, or 1% (*v*/*v*) limonene (NP BZC+LIM 0.5%_P407, NP BZC+LIM 0.75%_P407, and NP BZC+LIM 1%_P407, respectively) on the viscoelastic properties of the hydrogel was evaluated using oscillatory measurements at varying temperatures (Figure 3). The main parameters analyzed were the elastic modulus (*G*′), viscous modulus (*G*′), and sol–gel transition temperature (*T*_sol–gel_).

As shown in Figure 3, the hydrogels have liquid-like characteristics, where the *G*′ values were lower than *G*^″^ (Table 1) at temperatures below 22.4°C for P407–30% and BZC_P407, 21.2°C for NP_CTL_P407, 22.5°C for NP_BZC_P407, 20.9°C for NP_BZC_P407_LIM 0.5%, 21.2°C for NP_BZC_P407_LIM 0.75%, and 19.7°C for NP_BZC_P407_LIM 1%. However, above these temperatures, the nanoformulations exhibited a soft-to-hard-gel behavior, as evidenced by the increase in the value of *G*′, which is higher than the value of *G*^″^. This transition phase is known as the sol–gel transition temperature (*T*_sol–gel_), which is characterized by a rapid increase in the *G*′ and *G*^″^ values until 24°C promptly followed by a plateau that corresponds to the gel phase. Considering the storage moduli (*G*′) behavior, all nanoformulations showed increased viscosity with increasing temperature (data not shown), underlining the differences between liquid and gel rheological properties. All nanoformulations produced here demonstrated thermosensitive behavior, and the addition of drug, nanoparticles loaded with or without BZC, and limonene did not significantly affect *T*_sol–gel_, suggesting that hydrogel structural organization was maintained after additive incorporation. These results are in agreement with previous Poloxamer-based thermoresponsive hydrogel studies [13, 40].

### 3.3. *In Vitro* Release Assays and Mathematical Modeling

**Figure 4:**
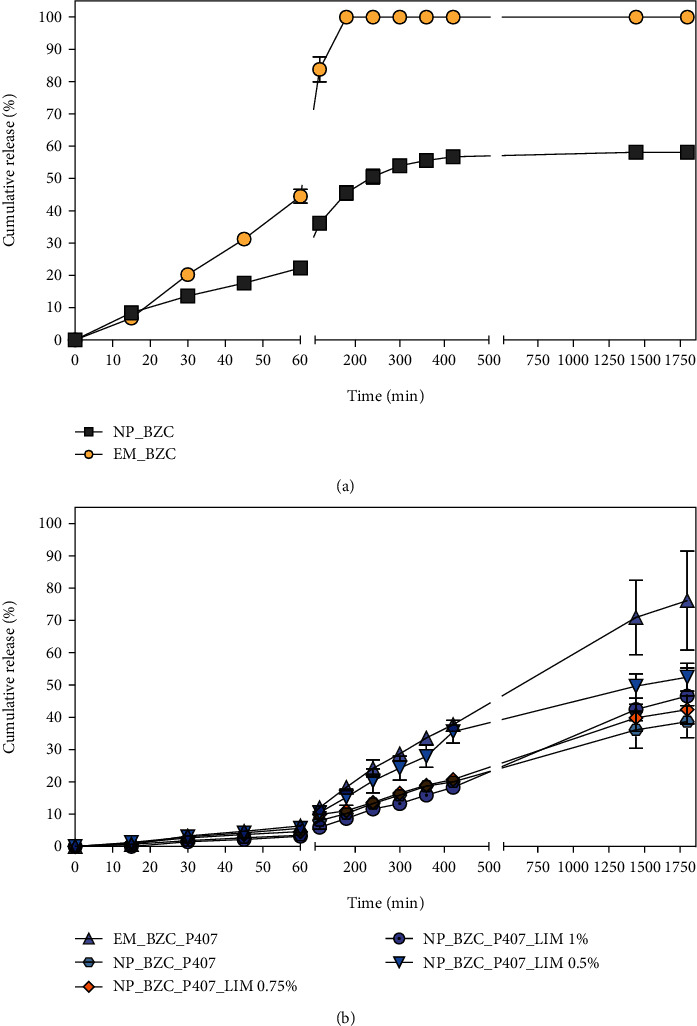
Kinetics of the release of BZC from suspension and hydrogel nanoformulations: (a) release profile of BZC nonencapsulated (EM_BZC) and BZC-loaded in PLGA nanoparticles (NP_BZC). (b) Release profile of BZC nonencapsulated incorporated in a Poloxamer P407 hydrogel (EM_BZC_P407), BZC-loaded in PLGA nanoparticles incorporated into a Poloxamer P407 hydrogel (NP_BZC_P407), and BZC-loaded in PLGA combined with limonene (NP_BZC_P407_LIM 0.5%, NP_BZC_P407_LIM 0.75%, and NP_BZC_P407_LIM 1%). The release assay was carried out using a vertical diffusion cell system at 32.5°C using a phosphate buffer solution at pH 7.4 as the acceptor medium. The analysis was performed in triplicate and quantified by HPLC.

The amount of BZC released from the PLGA nanoparticles (NP_BZC) and BZC-loaded nanoparticles incorporated into Poloxamer P407 hydrogels (EM_BZC_P407 and NP BZC_P407) and in combination with limonene (NP_BZC_P407_LIM 0.5%, NP_BZC_P407_0.75%, and NP_BZC_P407_1%) was studied using the Franz diffusion cell setup under sink conditions, and the results are shown in Figure 4.

In order to evaluate the release of nonencapsulated BZC, the drug was emulsified with PVA 0.3%, which was the same concentration used for nanoparticle preparation, due to the poor aqueous solubility of BZC (0.66 mg/mL) [42]. According to Figure 4(a), it was possible to observe a fast release of BZC when this drug was emulsified with PVA (yellow dots) which resulted in 99.5 ± 0.12% of the drug being released within 180 min. When this emulsified BZC was incorporated into Poloxamer-based hydrogels (Figure 4(b), purple dots), it achieved a sustained-release profile, with a significant reduction in the drug release percentage (*P* ≤ 0.05), where only 18 ± 1.4% of the BZC was released within 180 min, which is 5-fold less than that of BZC emulsified with PVA. This nanoformulation had a release rate of 76 ± 2.6% after 30 h.

The release profile of BZC-loaded PLGA nanoparticles (grey square) showed two distinct patterns: a fast initial release, observed as a release rate of 36 ± 1.9% within 90 min, followed by a more sustained release with a release rate of 56 ± 1% after 420 min both followed by a plateau. As observed for emulsified BZC, when PLGA nanoparticles loaded with BZC were incorporated into Poloxamer-based hydrogels, there was a significant reduction (*P* ≤ 0.05) in the BZC release rate. This hybrid system (nanoparticles and hydrogel) significantly decreased, approximately 6-fold, the initial rapid release of BZC during the first 90 min, which was followed by an increased sustained release over time, and the 38 ± 5% maximum release rate for this nanoformulation was observed only after 30 h. Among all nanoformulations tested, this nanoformulation showed the shortest release rate over time.

To evaluate the effect of limonene incorporation on BZC release, we added different concentrations of this compound to the hydrogel matrix. As shown in Figure 4 (blue triangle), limonene gradually increased the release rate of BZC, when compared to the hybrid system without this compound. Up to 90 min, there was no significant difference in the release rate of BZC in comparison to NP BZC_P407; however, after this period, there was a significantly higher release of BZC combined with limonene. After 30 h, the obtained release rate was 52 ± 4.3%, which is 1.4-fold higher than NP BZC_P407. Of note, further increases in limonene concentration (0.75 and 1%) did not cause significant differences in the BZC release rate when compared with a BZC-loaded hydrogel in the absence of limonene.

Several other studies have studied the association of polymeric nanoparticles and hydrogels in order to understand the drug delivery release profiles for topical applications. Poly(*ε*-caprolactone) nanoparticles loaded with a eutectic mixture of lidocaine and prilocaine were incorporated into Carbopol hydrogels, which resulted in sustained-release profiles for both Las, when compared to nonencapsulated drugs and nanoparticles alone, and also increased anesthetic efficacy *in vivo* [14]. In another study, lidocaine and prilocaine were coloaded into nanostructured lipid carriers (NLCs), which were then incorporated into xanthan hydrogels. There was an increased release profile of 10 h to deliver the total amount of anesthetic when the NLC was combined with the hydrogels [43]. Thus, together with the results obtained in the present work, it is possible to conclude that hybrid systems are an excellent alternative to prolong the release and consequently the anesthetic activity of different drugs.

The release profiles were evaluated using different mathematical models (zero-order, first-order, Higuchi, and Korsmeyer-Peppas). Linear regression was employed to calculate the release constant (*k*), correlation coefficient (*r*^2^), and release exponent (*n*). The results are summarized in Table S1.

As shown in Table S1, for nonencapsulated BZC (EM_BZC), the best fit to the release profile was obtained using the Higuchi model, which showed a higher coefficient of correlation (*r*^2^ ≥ 0.99) than the other models when applied. This model states that BZC liberation occurs via a diffusion process based on Fick's law. However, after the incorporation of EM_BZC into Poloxamer hydrogels, there was a significant decrease in drug release, which was also reflected in the release profile, better described by the zero-order model with a correlation coefficient of 0.9950. This model describes nanoformulations that release the same amount of drug per unit of time, independent of the drug concentration, leading to minimal fluctuations in drug levels. When BZC was loaded into PLGA nanoparticles, the release profile best fit the Korsmeyer-Peppas model, with a correlation coefficient of 0.9806 and a release exponent (*n*) value of 0.58, which suggests that the release mechanism corresponds to an anomalous diffusion mechanism (non-Fickian transport) that depends on both diffusion and relaxation (erosion) of the polymer matrix [44]. Similar results were found for the naproxen-loaded chitosan nanoparticles that were further incorporated into Poloxamer hydrogels and followed the Korsmeyer-Peppas model via an anomalous diffusion mechanism [40].

After incorporation of BZC-loaded nanoparticles into the hydrogels, the release mechanism best fits the Higuchi model, indicating that the main mechanism of BZC release is the diffusion process based on Fick's law [45]. Interestingly, when limonene was added to the hydrogel at concentrations of 0.5 and 1%, together with BZC-loaded nanoparticles, the release profiles fit well to both the Korsmeyer-Peppas and zero-order (*r*^2^ ≥ 0.99) models. These results suggest that the release of BZC occurs slowly and is not dependent on its concentration when combined with its diffusion and relaxation of the polymer chains of both the nanoparticles and hydrogel matrix. On the other hand, hydrogels containing BZC-loaded nanoparticles with 0.75% limonene fit better to the Higuchi model, where diffusion is dependent on Fick's law which governs the release. There was no significant difference in the release constant (*k*) when compared with hydrogels containing only BZC-loaded nanoparticles. Similar results were reported by Akkari et al., where the delivery of ropivacaine from a binary hydrogel nanoformulation (PL407-PL188) followed the Higuchi model [46].

### 3.4. *In Vitro* Permeation Studies across Strat-M® Artificial Membrane: Skin Model Permeation

**Figure 5:**
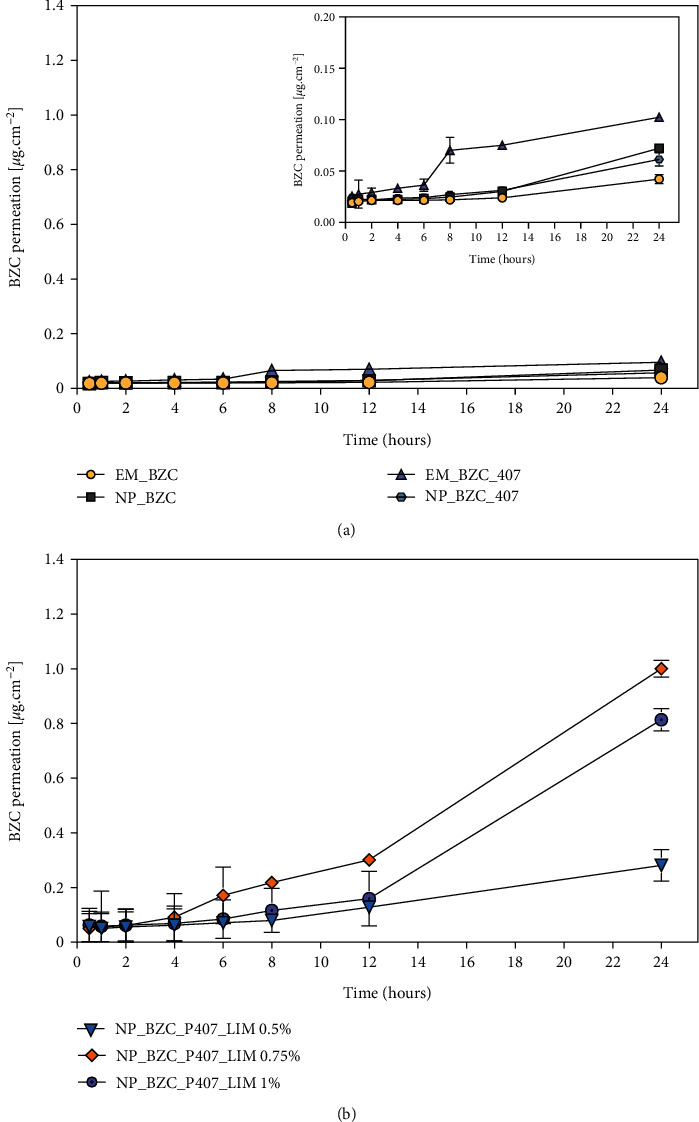
Permeation profile of BZC from different nanoformulations. The permeation assay was performed using a vertical diffusion cell at 32.5°C, in a phosphate buffer solution of pH 7.4. (a) EM_BZC and NP_BZC in suspension and incorporated into Poloxamer hydrogels. The inset represents the same permeation curve on a smaller *y*-axis scale. (b) NP_BZC incorporated into a Poloxamer hydrogel in combination with 0.5, 0.75, and 1% limonene (NP_BZC_P407_LIM 0.5%, NP_BZC_P407_LIM 0.75%, and NP_BZC_P407_LIM 1%), respectively.

The BZC permeation profiles from different nanoformulations were analyzed under sink conditions, and the results were plotted in terms of cumulative BZC permeation as a function of time (Figure 5). The slope of the permeation curve (linear portion) for the period of 0.5–8 h represents the permeation flux of BZC through the membrane, and the intersection with the *x*-axis shows the time necessary for the initiation of the permeation (lag time), as shown in Table 2.

According to Figure 5(a), a faster permeation rate (0.1 ± 0.03 *μ*g.cm^2^) was observed for the BZC nonencapsulated hydrogel (EM-BZC_P407) after 24 h, whereas BZC in emulsion (EM_BZC) had a permeation rate of 0.04 ± 0.003 *μ*g·cm^2^ for the same period. This increase in drug permeation after incorporation into the Poloxamer hydrogel could be explained by better formulation spreadability on the membrane surface, as observed by the rheological analysis, resulting in increased permeation rates. On the other hand, for NP_BZC, the amount of BZC which permeated after 24 h was 0.07 ± 0.002 *μ*g·cm^2^, and no significant differences were observed in the permeation profiles when these nanoparticles were incorporated into the hydrogels, which resulted in a permeation rate of 0.06 ± 0.006 *μ*g·cm^2^ during the same period. The results found here differ from those observed by Grillo et al. [13] who evaluated the permeation of BZC from plain hydrogels (P407 20%) or BZC-loaded poly(*ε*-caprolactone) nanoparticles incorporated into hydrogels and found lower permeation rates for BZC for hybrid systems (nanoparticles and hydrogels) as compared to the plain hydrogels. This can be attributed to the nanoparticle composition, considering PCL is the main component. In this study, we use a more complex hybrid system comprised of chitosan coating and its association with PLGA. Chitosan has gained attention as a coating for nanodrugs because it promotes satisfactory control of drug delivery in acidic conditions as it dissolves well at a pH of approximately 5 [47]. In addition, studies have shown that chitosan can interact with various lipids through hydrogen bonding, which results in the formation of a chitosan layer on the lipid layer by surface pressure [48, 49]. Considering that Strat-M membranes are impregnated with different lipids on their surfaces, the CS-coated PLGA nanoparticles could facilitate deposition on the membrane surface and facilitate drug permeation [13, 47].

**Figure 6:**
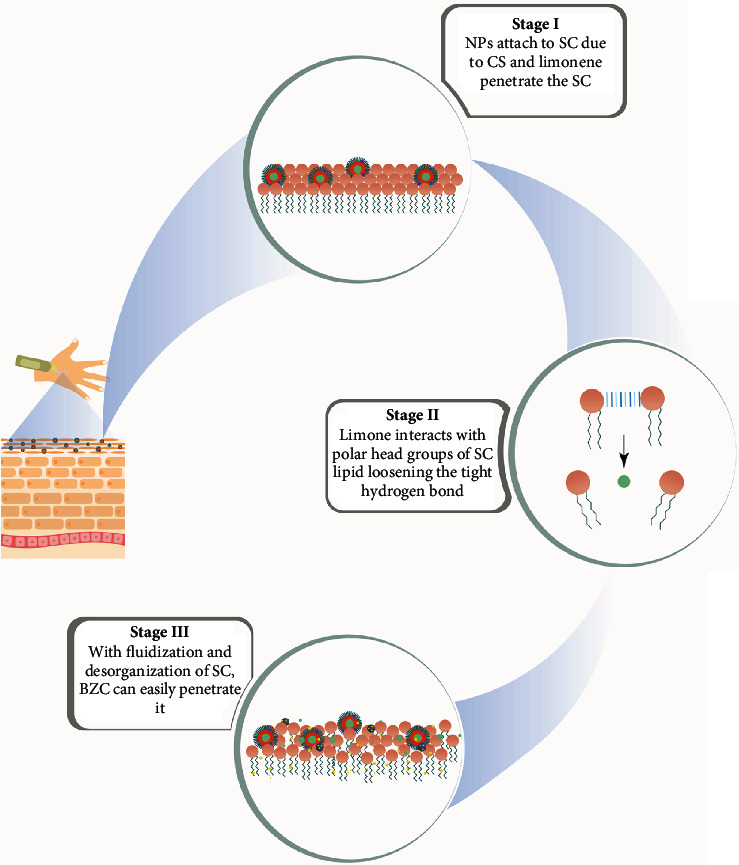
Schematic representation of the role of limonene in enhancing benzocaine penetration through the stratum corneum.

It is already known that essential oils and their volatile components can penetrate the skin and increase the penetration of different types of drugs from topical nanoformulations into deeper skin layers [50]. This work examined the capacity of limonene to increase the permeation of BZC in hybrid systems (nanoparticles and hydrogel) at three different concentrations (0.5, 0.75, and 1% *v*/*v*) (Figure 6(b)). As expected, there was a significant increase in BZC permeation at all the limonene concentrations tested. At the lowest concentration tested, there was a 5-fold increase in the BZC permeation rate compared to NP_BZC_P407 in the same period (24 h), and further increases in limonene concentration led to significant increases in BZC permeation. After 24 h, the permeation was 1.07 ± 0.03 *μ*g·cm^2^ and 0.87 ± 0.04 *μ*g·cm^2^ for hydrogels containing 0.75 and 1% *v*/*v* of limonene, which is 17-fold and 14.5-fold higher when compared to NP_BZC_P407 in the same time period, respectively.

Terpenes can effectively enhance the penetration of both hydrophilic and lipophilic drugs; however, some of them are better at enhancing the permeation of hydrophilic drugs due to their ability to bind hydrogen. Limonene is a hydrocarbon terpene that enhances the permeation of lipophilic drugs [51]. Both the lipophilicity of the molecule to be permeated and that of the permeation enhancer agent are important for enhancing the permeation of a drug across the skin. In our study, limonene in combination with the BZC-loaded hydrogel exhibited high enhancement of the flux and permeability coefficients, as well as a decreased lag time of the drug. The enhanced permeation provided by limonene can be attributed to its higher thermodynamic activity in the hydrogel matrix due to solubility changes [52].

Indeed, increased permeation using limonene as a penetration enhancer has been reported for topical and transdermal delivery. Patel et al. studied the transdermal delivery of raloxifene-encapsulated solid lipid nanoparticles in combination with Carbopol hydrogels with 5, 10, and 15% (*v*/*v*) limonene [53]. They found that 10% limonene had superior permeation flux compared to hydrogels without limonene or with other penetration enhancers, such as oleic acid [53]. Charoenputtakun et al. studied the dermal delivery of all-trans-retinoic acid-loaded solid lipid nanoparticles associated with terpenes (limonene and 1,8 cineole) as penetration enhancers and demonstrated that nanoparticles associated with 10% limonene resulted in the highest skin permeation and drug flux when compared to other nanoformulations and drug suspensions [54]. Together with these previous studies, we conclude that limonene is a good candidate for enhancing the topical and/or transdermal delivery of a wide variety of drugs. Multiple possible mechanisms are involved in the penetration enhancement by limonene, including increased solubility of the drug according to the lipid-protein partitioning principle within the stratum corneum layer, which may also increase the permeability of the drug, disturb the intracellular lipids of the stratum corneum due to extraction or fluidization (Figure 6), and alter conformations inside the keratinized protein component [24, 54].

Similarly, for the permeation rate, all other parameters calculated, such as flux, permeability coefficient, and lag time, were found to be of the same order: EM_BZC_P407 had a lower lag time than NP_BZC which had a lower lag time than NP_BZC_P407 and EM_BZC. For the hydrogels containing limonene, NP_BZC_P407_LIM 0.75% had the lowest lag time followed by NP_BZC_P407_LIM 1% and then NP_BZC_P407_LIM 0.5%. Both the nanoencapsulation and addition of limonene to the hydrogels showed increased drug permeation when compared to the nonencapsulated drug, in contrast to that observed in the release profile, where the nonencapsulated BZC was delivered faster. This could be attributed to the presence of polyolefin layers on the Strat-M® membrane surface, mimicking the epidermis lipid matrix [55, 56], which is absent from the cellulose acetate membrane composition used for the release assays. Even considering the differences among the BZC-hybrid system compositions (nanoparticles and hydrogels), increased BZC permeation parameters have been observed in previous reports [13, 57].

### 3.5. *In Vitro* Cell Toxicity

**Figure 7:**
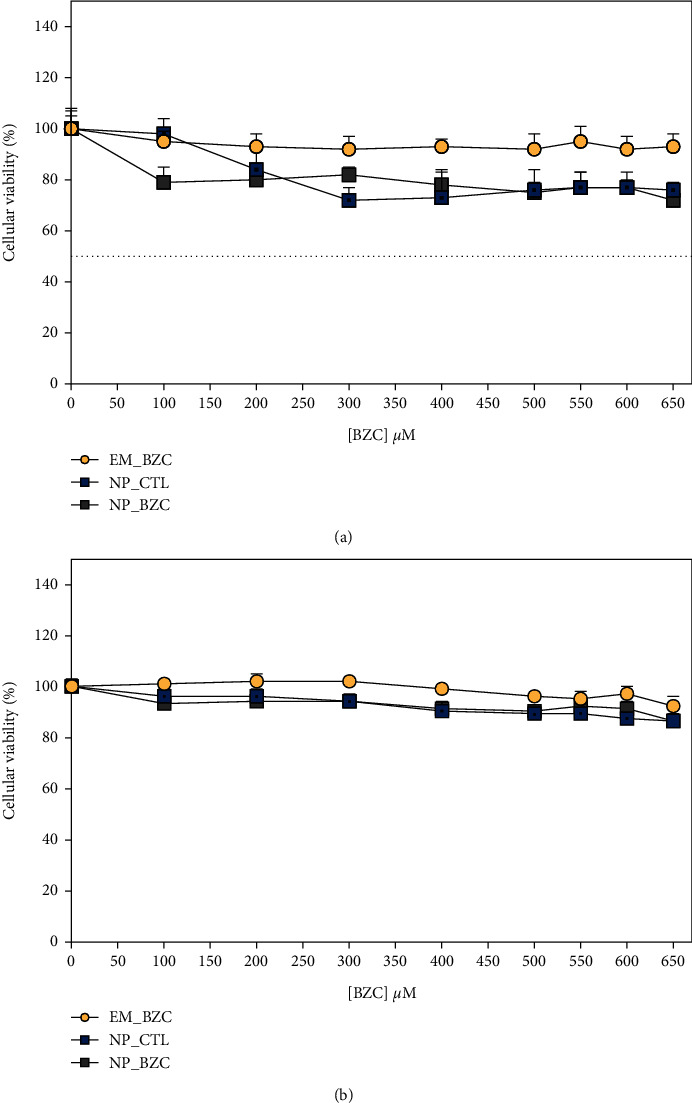
*In vitro* cytotoxicity of nonencapsulated BZC (EM_BZC), control nanoparticles (NP_CTL), and BZC-loaded nanoparticles (NP_BZC) tested by evaluating (a) keratinocyte (HaCaT) and (b) fibroblast (3T3) cell viability. By MTT assay. This data represents the mean ± SD, of three determinations (*n* = 6).

*The in vitro* cytotoxicity of nonencapsulated BZC (EM_BZC) and PLGA nanoparticles (NP_CTL and NP_BZC) was tested by evaluating HaCaT (Figure 7(a)) and 3T3 (Figure 7(b)) cell viability using the MTT assay. According to our results, it is possible see that all treatments were slightly more toxic to keratinocytes when compared to fibroblasts, at the tested concentration ranges. After 24 h, the keratinocyte cell viability (HaCaT) for the cells treated with EM-BZC was 93 ± 5%, in contrast to 76 ± 3% and 72 ± 3%, for the cells treated with NP_CTL and NP_BZC, respectively. It seems that this small decrease in viability for cells treated with both nanoparticles is due to the characteristics of the nanoparticles themselves and not to the BZC addition since there were no significant differences in the viability of cells treated with NP_BZC compared to those treated with NP_CTL. For the 3T3 cells (Figure 7(b)), the exposure to EM_BZC, NP_CTL, and NP_BZC caused slight changes in the cell viability, with values at 92 ± 4%, 86 ± 3%, and 86 ± 2%, respectively, after 24 h of treatment. Nevertheless, the results indicate that there is a concentration-dependent mortality for both cell lines at 24 h which fall below 72% for HaCaT and 86% for 3T3; both values are in accordance with the recommended guidelines for biomedical devices and delivery systems (DIN EN ISO 10993-5) which indicate these types of materials can be considered noncytotoxic at ≥70% cell viability after exposure [58, 59]. Thus, the results obtained in the current study show that PLGA nanoparticles are generally safe. Similar results have been found for solid lipid nanoparticles and poly(*ε*-caprolactone) nanocapsules loaded with ATC, poly(*ε*-caprolactone) nanospheres loaded with lidocaine, PLGA nanospheres loaded with ropivacaine, and alginate nanoparticles loaded with bupivacaine, where LA encapsulation resulted in acceptable cellular viability [2, 59–61].

## 4. Conclusions

BZC-loaded PLGA nanoparticles demonstrate good physicochemical properties, high encapsulation efficiency, spherical shape, and stability as a function of the storage time (90 days). The nanoparticles caused a slight decrease in the viability of keratinocytes and fibroblasts, which is acceptable according to the guidelines for the evaluation of the cytotoxicity of biomedical devices and delivery systems. Both nanoparticles and hydrogels were able to delay the release of benzocaine compared to the nonencapsulated drug. In contrast, there was an increase in the permeation rates when the drug was encapsulated in nanoparticles and hydrogels compared to nonencapsulated benzocaine. Furthermore, in association with the bioadhesive properties of chitosan, the addition of limonene into hydrogels containing BZC-loaded nanoparticles resulted in higher permeation rates, indicating that limonene is a good candidate for drug penetration enhancement. These results demonstrated that the hybrid nanoformulations could be a promising drug delivery system for LAs, prolonging anesthetic efficacy and decreasing toxicity; however, further studies evaluating the biological activity after topical application should be conducted.

## Figures and Tables

**Table 1:** Rheological parameters of P407 hydrogels as a function of frequency (1 Hz) measured at 32.5°C. The parameters analyzed were the elastic (*G*′) and viscous (*G*^″^) moduli, viscosity (*ƞ*), and sol to gel transition temperature (*T*_sol–gel_).

Sample	*G*′ (Pa)	*G* ^″^ (Pa)	*G*′/*G*^″^	*η* (×10^6^ mPas·s)	*T*_sol–gel_ (°C)
P407 30%	14270	641.9	22.2	2.27	22.4
BZC_P407	16370	767.2	21.8	2.61	22.4
NP CTL_P407	13710	567.1	24.2	2.18	21.2
NP BZC_P407	12680	579.7	21.9	2.02	22.5
NP BZC+LIM 0.5%_P407	12850	497.1	25.8	2.05	20.9
NP BZC+LIM 0.75%_P407	14440	531	27.2	2.30	21.2
NP BZC+LIM 1%_P407	13330	378.5	35.2	2.12	19.7

**Table 2:** Permeation parameters of BZC across Strat-M® artificial membranes from emulsions (EM_BZC), BZC-loaded PLGA nanoparticles (NP_BZC), emulsion incorporated into Poloxamer P407 hydrogels (EM_BZC_P407), BZC-loaded PLGA nanoparticles incorporated into Poloxamer P407 hydrogels (NP_BZC_P407), and BZC-loaded PLGA nanoparticles incorporated into P407 hydrogels associated with 0.5, 0.75, and 1% limonene (NP_BZC_P407_LIM 0.5%, NP_BZC_P407_LIM 0.75%, and NP_BZC_P407_LIM 1%), respectively.

Nanoformulation	Flux (% cm^−2^·h^-1)^	Permeability coefficient (cm·h^−1^)	Lag time (h)
EM_BZC	2.7 × 10^−4^	1.13 × 10^−7^	73.3
NP_BZC	5.2 × 10^−4^	2.20 × 10^−7^	39.1
EM_BZC_P407	4.8 × 10^−3^	8.11 × 10^−6^	4.01
NP_BZC_P407	5.0 × 10^−4^	8.40 × 10^−7^	43.9
NP_BZC_P407_LIM 0.5%	3.6 × 10^−3^	6.09 × 10^−6^	14.8
NP_BZC_P407_LIM 0.75%	2.4 × 10^−2^	4.11 × 10^−5^	1.11
NP_BZC_P407_LIM 1%	7.3 × 10^−3^	1.21 × 10^−5^	7.53

## Data Availability

The data that support the findings of this study are available on request from the corresponding authors.

## Supplementary Materials

Supplementary Materials Figure S1: nanoparticle concentration and size distribution measured by nanoparticle tracking analysis (NTA): (a) control nanoparticles (NP_CTL) time 0 (green lines) and after 90 days (orange lines) and (b) BZC-loaded nanoparticles (NP_BZC) time 0 (blue lines) and after 90 days (red lines). Table S1: mathematical model parameters, such as release constant (*k*), correlation coefficient (*r*), and release exponent (*n*) after application of zero-order, first-order, Higuchi, and Korsmeyer-Peppas models to the release curve of BZC from different formulations.
