# Supplementary material for: Using Chitosan-Coated Polymeric Nanoparticles-Thermosensitive Hydrogels in association with Limonene as Skin Drug Delivery Strategy
Source: Biomed Res Int. 2022 Apr 7;2022:9165443. doi: 10.1155/2022/9165443 (PMC9010220; doi:10.1155/2022/9165443)
Supplement: Supplementary Materials — Figure S1: nanoparticle concentration and size distribution measured by nanoparticle tracking analysis (NTA): (a) control nanoparticles (NP_CTL) time 0 (green lines) and after 90 days (orange lines) and (b) BZC-loaded nanoparticles (NP_BZC) time 0 (blue lines) and after 90 days (red lines). Table S1: mathematical model parameters, such as release constant (k), correlation coefficient (r), and release exponent (n) after application of zero-order, first-order, Higuchi, and Korsmeyer-Peppas models to the release curve of BZC from different formulations. [file 9165443.f1.docx]

**SUPPLEMENTARY MATERIAL**

**Figure S1:** Nanoparticles concentration and size distribution measured by Nanoparticle Tracking Analysis (NTA). **A)** control nanoparticles (NP_CTL) time 0 (green lines) and after 90 days (orange lines) and **B)** BZC-loaded nanoparticles (NP_BZC) time 0 (blue lines) and after 90 days (red lines).

**Table S1:** Mathematical model parameters, such as release constant (k), correlation coefficient (r) and release exponent (n) after application of Zero order, first order, Higuchi and Korsmeyer-Peppas models to the release curve of BZC from different formulations.

| **Parameter** | **Zero Order** | **First Order** | **Higuchi** | **Korsmeyer-Peppas** |  |  |  |  |
| --- | --- | --- | --- | --- | --- | --- | --- | --- |
| **EM_BZC** | | | | |  |  |  |  |
| Release Constant (k) | 0.5730 min^-1^ | 5.9 x 10^-3^ min^-1^ | **10.29 min^-1/2^** | 1.29 min^-1^ |  |  |  |  |
| Correlation Coefficient (r)  Release exponent (n) | 0.9639  - | 0.7610  - | **0.9912**  **-** | 0.9688  1.07 |  |  |  |  |
| **EM_BZC_P407** | | | | |  |  |  |  |
| Release Constant (k) | **0.09175 min^-1^** | 3.3 x10^-3^ min^-1^ | 2.25 min^-1/2^ | 0.35 min^-1^ |  |  |  |  |
| Correlation Coefficient (r)  Release exponent (n) | **0.9950**  **-** | 0.7539  - | 0.9880  - | 0.9652  1.10 |  |  |  |  |
| **NP_BZC** | | | | | - | - | - | 1.10 |
| Release Constant (k) | 0.1215 min^-1^ | 1.7 x10^-3^ min^-1^ | 3.12 min^-1/2^ | **1.58 min^-1^** |  |  |  |  |
| Correlation Coefficient (r)  Release exponent (n) | 0.8871  - | 0.7634  - | 0.9677  - | **0.9806**  **0.58** |  |  |  |  |
| **NP_BZC_P407** | | | | |  |  |  |  |
| Release Constant (k) | 0.04906 min^-1^ | 3.0 x10^-3^ min^-1^ | **1.2 min^-1/2^** | 0.35 min^-1^ |  |  |  |  |
| Correlation Coefficient (r)  Release exponent (n) | 0.9875  - | 0.7990  - | **0.9920**  **-** | 0.9891  0.99 |  |  |  |  |
| **NP_BZC + LIM 0.5%_P407** | | | | | - | - | - | 0.84 |
| Release Constant (k) | **0.08042 min^-1^** | 3.0 x10^-3^ min^-1^ | 1.95 min^-1/2^ | **0.44 min^-1^** |  |  |  |  |
| Correlation Coefficient (r)  Release exponent (n) | **0.9962**  **-** | 0.8466  - | 0.9705  - | **0.9965**  **0.95** |  |  |  |  |
| **NP_BZC + LIM 0.75%_P407** | | | | |  |  |  |  |
| Release Constant (k) | 0.04793 min^-1^ | 2.5 x10^-3^ min^-1^ | **1.19 min^-1/2^** | 0.50 min^-1^ |  |  |  |  |
| Correlation Coefficient (r) | 0.9783 | 0.7920 | **0.9933** | 0.9849 |  |  |  |  |
| Release exponent (n) | - | - | **-** | 0.84 |  |  |  |  |
| **NP_BZC + LIM 1%_P407** | | | | |  |  |  |  |
| Release Constant (k) | **0.04400 min^-1^** | 2.9 x10^-3^ min^-1^ | 1.08 min^-1/2^ | **0.39 min^-1^** |  |  |  |  |
| Correlation Coefficient (r) | **0.9942** | 0.8724 | 0.9888 | **0.9956** |  |  |  |  |
| Release exponent (n) | **-** | - | - | **0.91** |  |  |  |  |
